# Revealing Nanoindentation Size-Dependent Creep Behavior in a La-Based Metallic Glassy Film

**DOI:** 10.3390/nano9121712

**Published:** 2019-12-01

**Authors:** Yi Ma, Yuxuan Song, Taihua Zhang

**Affiliations:** 1 College of Mechanical Engineering, Zhejiang University of Technology, Hangzhou 310014, China; songyux@zjut.edu.cn; 2 Institute of Solid Mechanics, Beihang University, Beijing 100191, China

**Keywords:** nanoindentation, creep, metallic glass, size effect, strain rate sensitivity

## Abstract

We systematically studied nanoindentation size effect on creep deformation in a La-based metallic glassy film, including holding depth effect and indenter size effect. Creep displacement was mainly dependent on both holding strain and deformation volume beneath indenter. Under elastic holding, creep strain was merely holding strain–dependent. While for plastic holding, creep strain was greatly enhanced by adopting smaller indenter and/or decreasing holding depth at the same holding strain. A strong nanoindentation size effect on creep resistance was validated. Strain rate sensitivities (SRS) were calculated, which were obviously higher at elastic regions than at plastic holdings. The relationship between SRS value and creep mechanism in metallic glass was discussed.

## 1. Introduction

Since the debut of metallic glass in 1960s [1], this new member of the glass family has attracted lots of attention for its excellent mechanical properties such as large elastic limit, high strength and strong wear resistance [2]. Metallic glasses’ own unique atomic structure, i.e., long-range disorder with short-range order, which endows a special importance to these materials in condensed matter physics [3]. In the last few decades, materials researchers have made significant efforts to develop different bulk metallic glass compositions and fabricate these alloys in large volume, in order to apply them at various engineering fields. Until now, the limited critical size and serious brittleness hindered the widespread commercial application of bulk metallic glasses [4]. In recent years, size effect of mechanical properties in material has been on the cutting edge of material science and micro/nano mechanics [5,6,7]. For metallic glass, both strength and ductility could be largely enhanced by reducing sample dimension down to the nanoscale [8,9,10]. Even localized shear banding, which is the typical deformation mechanism in metallic glasses at low temperatures, could be avoided as sample size reduces down to about 100 nm, indicating a deformation mode transition [11,12]. Hence metallic glass is expected to be a good candidate in the field of nano/micro-electromechanical systems and nano-devices, combined with excellent shaping ability (around *T*_g_) and defect-free structure [13,14,15].

Creep resistance is an important mechanical parameter of engineering materials [16], particularly for those structures bearing high temperature and/or long-term stress. The conventional creep behavior of metallic glass is not fully investigated by uniaxial testing due to its limited size and poor processability in fabricating standard specimen. By the means of nanoindentation technology, mechanical properties could be probed in a micro region on the surface of flat specimen. The local time-dependent plastic deformation could be precisely and conveniently traced under nanoindention. In order to reduce the influence of thermal drift, nanoindentation creep measurement is commonly performed at room temperature. The sample size effect on creep deformation of metallic glasses has also been examined by nanoindentation. Yoo et al. reported an enhancement of creep flow in a Zr-based metallic glassy nanopillar by flat indenter as specimen diameter decreased from 2000 to 250 nm [17]. Wang et al. investigated creep behaviors of Cu-Zr films with thickness from 1000 to 3000 nm [18]. Ma et al. reported thickness effects on the creep deformation in Cu-Zr-Al and Ni-Nb metallic glassy films [19,20]. According to previous results, metallic glasses with smaller dimensions exhibit more pronounced creep deformation under both elastic and plastic holdings. In other words, creep behavior of metallic glass is against the rule of “smaller is stronger”.

Relying on nanoindentation, indentation size effect on creep behavior could also be studied at various holding depths. In this scene, the specimen dimension and intrinsic structure state is constant whilst creep behavior could be correlated with deformation zone beneath the indenter. The correlation between creep deformation and holding depth has also been widely reported in metallic glasses, where creep displacement was gradually increased with increasing holding depth [21,22,23,24]. Qualitatively, more excess free volume content generated at deep holding depths was generally suggested as the reason. However, the previous nanoindentation characterization of creep deformation and explanation could be a little doubtable and may have missed the details of creep behavior. For the commonly used Berkovich nanoindentation, the holding strain is unchanged at various holding depths, and it is reasonable to observe that creep displacement is increased with increasing holding depth due to the accordingly enlarged deformation volume. Further, there is no direct evidence for the holding depth-correlated free volume content under the similar strain in metallic glasses. Basically, holding strain or stress is the most crucial testing condition on the creep behavior of a material at a fixed temperature using conventional methods. On account of this, the indentation size effect on creep behavior should be examined based on the deformation volume and holding strain. With this in mind, we conducted nanoindentation load-holding tests under spherical tips of various radii, by which the holding strain effect on creep deformation could be systematically investigated. In comparison, a standard Berkovich indenter was also adopted to probe creep deformation at the same holding depths. For the nanoindentation tests, a La-based metallic glassy film with low creep resistance was selected [25]. The smooth and uniform surface of metallic glassy film can ensure nanoindentation accuracy at shallow depths [26].

## 2. Materials and Methods

For the sputtering target, La_60_Co_20_Al_20_ (nominal composition in at.%) alloy was prepared by high-vacuum casting from high purity (99.99 wt.%) elements. A 2-inch clean silicon wafer was adopted as substrate during magnetron sputtering. Before deposition, the base pressure of the chamber was kept below 5 × 10^−7^ Torr to avoid oxidation. The target-to-substrate distance was kept constant, equal to 100 mm. The working pressure of argon atmosphere was set about 1 mTorr and the power on target was fixed as 150 W in a direct current (DC) mode during the deposition. The deposition duration was 120 minutes. The film thickness can be directly measured from cross-sections by scanning electron microscope (SEM), which was 2.7 μm [27]. The chemical composition of the as-prepared film was La_55_Co_20_Al_25_, by means of *X*-ray energy dispersive spectrometer (EDS) attached to the SEM. The amorphous nature of the film has been confirmed by *X*-ray diffraction (XRD) with Cu Kα radiation in a previous work [25].

The nanoindentation creep tests were conducted at constant temperature of 20 °C on Agilent Nano Indenter G200. A constant-load holding method was adopted, during which displacement of indenter into the surface at a prescribed load could be continuously recorded. The indenter was held for 500 s at maximum depths of 20, 40, 60, 90, 150, 250 and 500 nm. The loading strain rate was fixed, equal to 0.05 s^−1^. A standard Berkovich indenter and three different spherical indenters with nominal radii of 1, 5 and 20 μm were used. Upon calibration on fused silica, the true contact radii of spherical tips were obtained as 0.6, 2.95 and 9.8 μm, respectively. Twenty-five independent measurements were performed under each testing condition. All the nanoindentation tests were carried out until thermal drift reduced to below 0.02 nm/s and drift correction was strictly performed at 10% of the maximum load during the unloading process.

## 3. Results and Discussion

**Figure 1:**
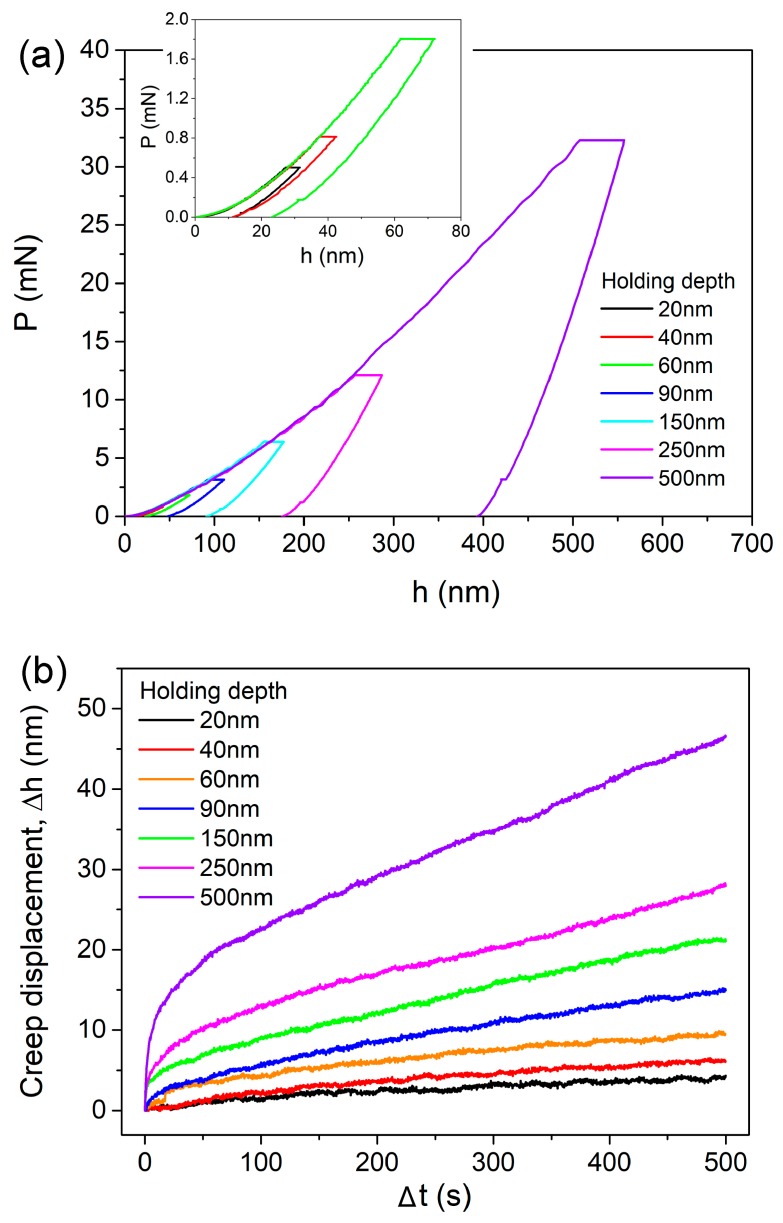
(**a**) Typical creep *P-h* curves at various holding depths under 2.95 μm spherical tip. *P-h* curves at shallow depths are enlarged in the inset. (**b**) Creep displacements at various holding depths were plotted with holding time.

Figure 1a exhibits the representative creep *P-h* curves with various holding depths under the spherical tip of 2.95 µm radius, as an illustration. The *P-h* curves at 20, 40 and 60 nm were enlarged, as shown in the inset. Clearly, permanent deformation occurred at holding stage, even for the 20 nm holding test. For nanoindentation measurement in the film-substrate system, the obtained elastic modulus and hardness would be largely influenced by substrate response once holding depth is beyond a critical value [28,29]. It should be mentioned that the “safe” depth for avoiding substrate effects in the metallic glassy film/silicon system could be larger than 20% of film thickness, according to author’s previous work [19]. Thus substrate effect on creep deformation could be negligible even at 500 nm holding. The corresponding creep displacements during holding stage were plotted as a function of holding time, as shown in Figure 1b. In order to observe creep deformations at different holding depths more intuitively, the onsets of displacement and time at holding stage were both set to be zero. For nanoindentation creep flow, the transient stage was much shorter (less than 30 s) than that of conventional creep and even disappeared at 20 and 40 nm holdings. Both the creep displacement and creep rate at steady-state stage were enhanced by increasing holding depth. It is worth mentioning that the surface roughness of La-Co-Al metallic glassy film was about 0.5 nm on the area of 5 × 5 μm in author’s previous work [26]. Therefore the surface roughness could be negligible to the holding depths used here.

**Figure 2:**
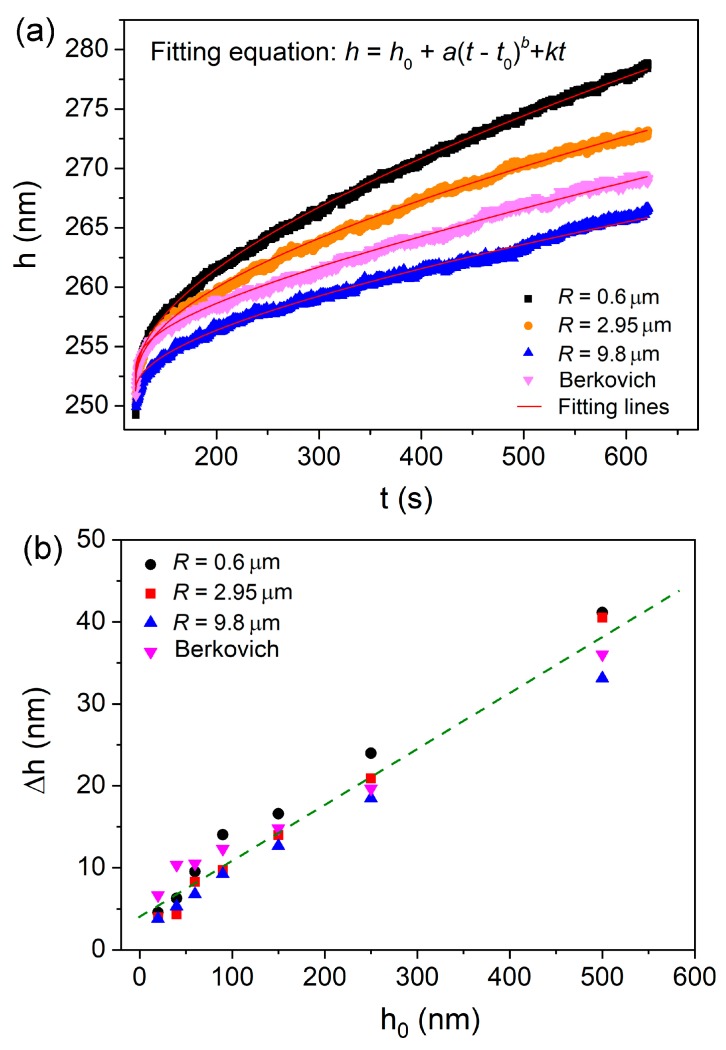
(**a**) Representative creep flow curves under spherical and Berkovich indenters at a holding depth of 250 nm, and they can be perfectly fitted by the empirical equation. (**b**) Mean creep displacements by four indenters were plotted with holding depth.

Figure 2a shows the representative creep curves for all the spherical and Berkovich indenters at 250 nm for comparison. The creep deformation was more pronounced under a smaller spherical tip. The creep flow by Berkovich indenter lay between those of the 2.95 and 9.8 µm spherical tips. All the creep curves could be perfectly fitted (*R*^2^ > 0.99) by an empirical law:

*h*(*t*) = *h*_0_ + *a*(*t* − *t*_0_)*^b^* + *kt* (1)

where *h*_0_*, t*_0_ are the displacement and time at the beginning of holding stage, *a*, *b*, *k* are the fitting constants. The total creep displacements at the end of holding stage by four indenters were recorded, which were plotted with holding depth in Figure 2b. The mean value was obtained from more than fifteen effective creep curves for each case, error bars were not shown for a clear view. Generally, total creep displacements were almost linearly increased with holding depth, and the increasing rate was apparently independent of indenter size. For a standard Berkovich indenter (without tip bluntness), the imposed plastic volume and stress distribution during nanoindentation are self-similar at various pressed depths. And the nanoindentation strain is constant, equal to ~7.1% (0.2cot70.5°). Ideally, creep displacement by Berkovich indenter in a homogeneous material should be in proportion to the holding depth, whilst creep strain would be invariable. While for a spherical indenter, the stress state of deformation zone evolves from elastic to elastoplastic with increasing pressed depth. And then the plastic strain continuously increases with pressed depth. As more severely plastic deformation occurs at deeper depths, more excess free volume and shear bands would be generated, causing better atomic mobility. Thus the increased creep displacement under spherical indenters could be attributed to the multiple effects of enlarged deformation volume, increased holding strain and more dramatic structural agitation. In previous reports, the holding depth-facilitated creep displacement was qualitatively explained by more excess free volume generated at deep holding depth [21,22,23,24]. Here we do not intend to deny previous conclusions, however the structure agitation would not be decisive to the change of creep displacement at various holding depths, particularly for Berkovich nanoindentation. The deformation volume and holding strain played more important roles on creep displacement under nanoindentation, which was similar to a conventional creep test.

**Figure 3:**
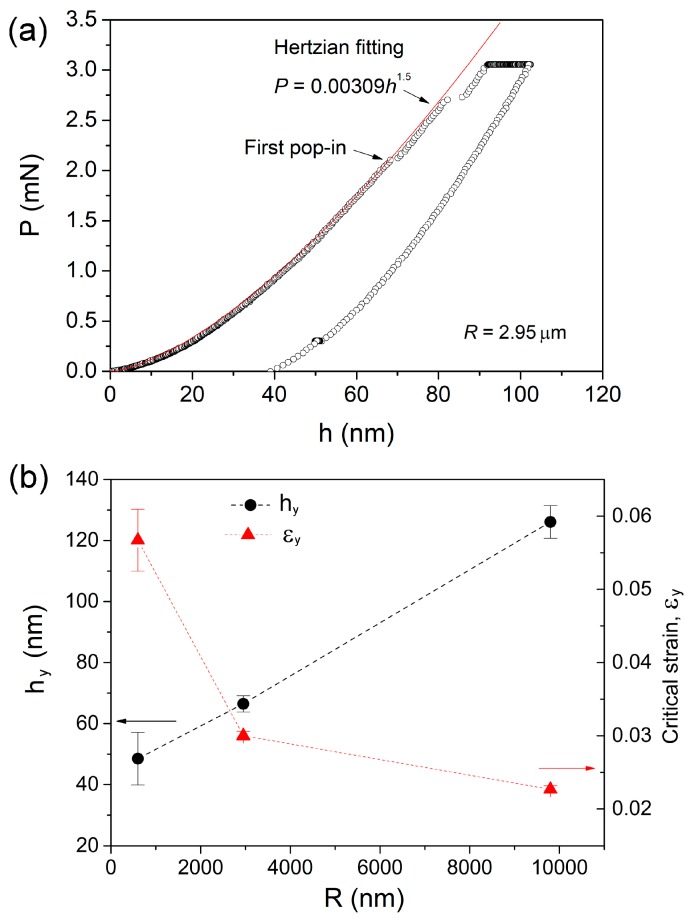
(**a**) Typical *P-h* curve under 2.95 μm spherical tip at pressed depth of 90 nm, the initial loading segment could be perfectly fitted by Hertzian contact theory. (**b**) The critical displacement and strain at the first pop-in event were plotted with spherical tip radius.

As Jang et al. reported that nanoindentation creep deformation was distinctly changed at elastic and plastic holdings by a spherical indenter [24]. It is necessary to distinguish the elastic and plastic holdings at various depths by the spherical tips. Figure 3a shows the typical creep *P-h* curve at 90 nm for 2.95 µm indenter. The pop-in events clear occurred, which indicated the generation of shear bands. At such shallow pressed depths, phase transformation or cracking in the silicon substrate could be excluded [30]. The initial loading sequence could be well-fitted by the Hertzian elastic contact equation [31], given by:

(2) $$P=\frac{4}{3}E_{r}\sqrt{R}h^{1.5}$$

where *E_r_* is the reduced elastic modulus and *R* is the tip radius. The elastic constant of the film can be deduced by:

(3) $$\frac{E_{s}}{1-v_{s}^{2}}={\left(\frac{1}{E_{r}}-\frac{1-v_{i}^{2}}{E_{i}}\right)}^{-1}$$

where *E* and v are the elastic modulus and Poisson’s ratio, with the subscripts *s* and *i* representing the sample and the indenter, respectively. For commonly used diamond tip, *E_i_* = 1141 GPa and v*_i_* = 0.07, it should be mentioned that the Hertzian fitting line just deviated from *P-h* curve at the position of first pop-in. This coincidence clearly indicates the transition from elastic to plastic deformation once the first pop-in emerges, which also could be regarded as the onset of yielding during nanoindentation. Figure 3b shows the critical displacements *h*_y_ at first pop-in events for three spherical tips, which was linearly increased with increasing tip radius. Accordingly, the minimum plastic holding depths were 60, 90 and 150 nm for spherical tips of 0.6, 2.95 and 9.8 µm radii, respectively. On the other hand, the nanoindentation strain at first pop-in can be estimated by $\varepsilon_{y}=0.2\left(\alpha /R\right)$, α is the contact radius equal to $\sqrt{Rh}$ for elastic contact. As exhibited in Figure 3b, the critical strains were 5.5%, 3.1% and 2.2% for spherical tips of 0.6, 2.95 and 9.8 µm radii, respectively. The elastic limit detected by 0.6 µm spherical tip was far beyond the typical ~2% for bulk metallic glasses. While under the 9.8 µm spherical tip, the estimated elastic limit reduced down to the conventional level. This strong indenter size effect on “elastic limit” could be mainly due to the complicated stress distribution beneath indenter. To form a shear band during nanoindentation, there needs a certain space along the shear path of which stress has been beyond the yield stress [32]. By this assumption, the “true” occurrence of yielding could be much earlier than the first pop-in event or incipient plasticity during loading sequence. Under the smaller tip, the larger gap of critical load or displacement between the “true” yielding point and the appearance of first pop-in could be conceived. As for 9.8 µm spherical nanoindentation, the stress distribution beneath the indenter is spacious enough. It is fairly easy to meet the critical requirement of space for generating a shear band once the maximum stress at a point reaches yield stress. Therefore, the enhancement of elastic limit under smaller spherical indenter can be explained by the delay of initial shear band.

**Figure 4:**
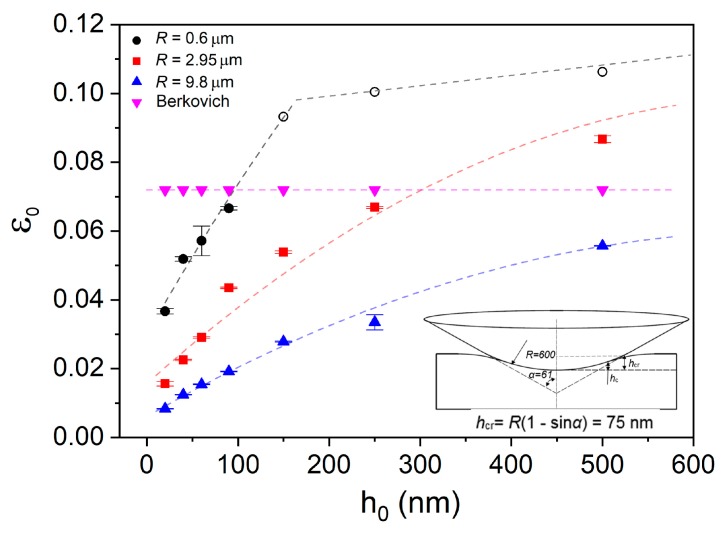
The corresponding nanoindentation holding strains at various holding depths for all indenters. For 0.6 μm spherical indenter, holding depth would exceed the boundary between conical body and spherical tip at 150, 250 and 500 nm.

**Figure 5:**
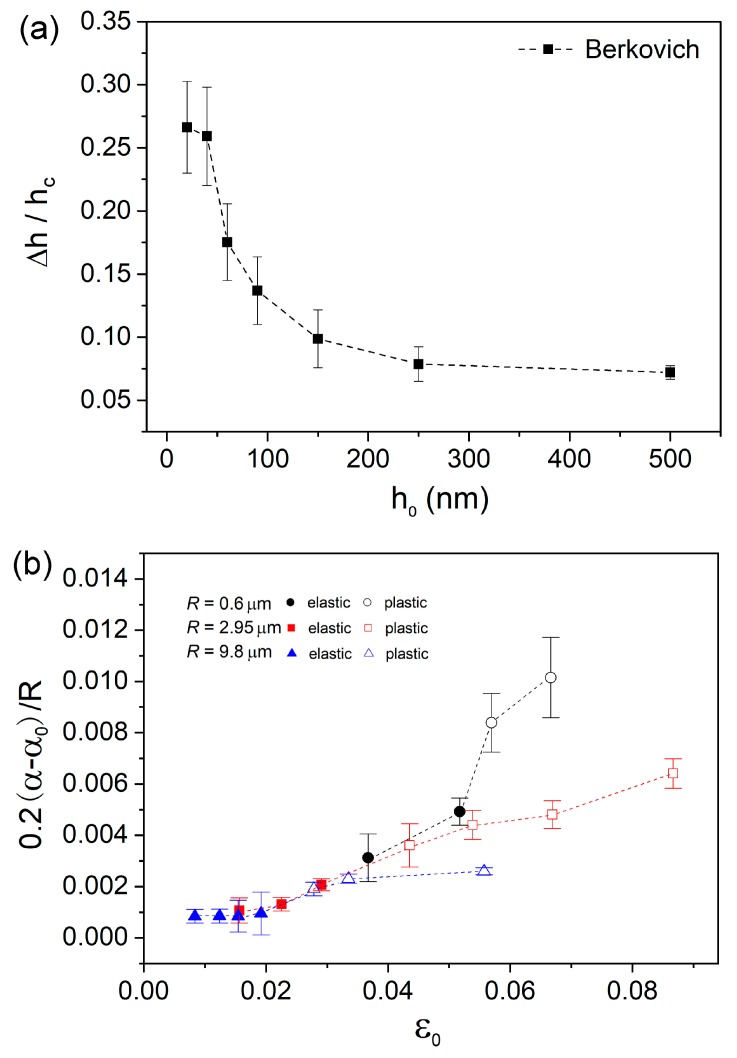
(**a**) Total creep strains under the Berkovich tip were plotted with holding depths. (**b**) Total creep strains under three kinds of spherical tips were plotted with initial strain before holding stage. The initial strains were divided as elastic and plastic strain by solid and hollow tags, respectively.

For the plastic contact under spherical tip, the contact radius α is $\sqrt{2Rh_{c}}$, where $h_{c}$ is the contact depth equal to *h_c_* = *h* − *ε* × *P/S*, *ε* = 0.75, *S* is the stiffness deduced from the unloading curve. The nanoindentation strains $\varepsilon_{0}$ for the four indenters at each holding depth were computed and exhibited in Figure 4. It should be mentioned that the Berkovich indenter used herein was treated as an ideal tip. In fact, nanoindentation strain beneath Berkovich indenter is gradually increased at shallow depth and then tends to be stable ~7.1% due to tip bluntness. The configuration of a spherical indenter is a conical body with spherical tip, as shown in the inset of Figure 5. Once the holding depth is beyond the upper boundary between conical body and spherical tip, the calculation formula for nanoindentation strain would be invalid. The “critical contact depth” *h*_cr_ at the boundary could be computed as: $h_{cr}=R\left(1-\sin61^{{}^{\circ}}\right)$. For 2.95 and 9.8 µm spherical indenters, the contact depths at all the adopted holding depths were below the “critical contact depth” of 370 and 1230 nm, respectively. In contrast, for the 0.6 µm spherical indenter, the contact depth at holding depth of 90 nm was just below the “critical depth” of 75 nm. Thus nanoindentation strains by the 0.6 µm spherical indenter at 150, 250 and 500 nm were unable to be precisely estimated. These points were marked by hollow tags and roughly assumed to be in the range of 9% to 11% (0.2cot61°). Clearly, the smaller the spherical tip radius, the faster the increase rate of initial holding strain with increasing holding depth.

Relying on the results of holding strains at various depths by spherical indenters, several details of creep displacements in Figure 2b could be explained. (i). Creep displacements by Berkovich indenter were higher than those by spherical tips at shallow depths as 20, 40 and 60 nm. The reason is that elastic deformation was dominating beneath spherical indenters at shallow depths, and the corresponding holding strains were much less than the ~7.1% of Berkovich nanoindentation. (ii). Creep displacements under 0.6 µm were higher than others at holding depths larger than 90 nm. It is reasonable that holding strain under 0.6 µm spherical indenter was quickly increased and exceeded the others at deep nanoindentations as shown in Figure 4. (iii). Creep displacement at a certain holding depth did not change much as changing indenter size. This is mainly due to the combined effects of deformation volume and holding strain. At the same holding depth, holding strain was lower under spherical indenter with larger radius. However, its deformation volume was apparently greater, which compensated the weakened effect on creep displacement by lower holding strain.

As it was revealed that creep displacement strongly relied on testing conditions, the non-dimensional creep strain could be adopted to represent creep resistance. For creep deformation under Berkovich indenter, we defined creep strain as Δ*h*/*h*_c_, in which Δ*h* is the total creep displacement and *h*_c_ is the contact displacement at the beginning of holding stage. Clearly, creep strain was rapidly decreased with holding depth and tended to be stable at deep positions, as exhibited in Figure 5a. That is to say, creep deformation was actually depressed with increasing holding depth under Berkovich indenter. This result confirms the previous reports about sample size-dependent creep flow. From the perspective of structure agitation, the density of shear bands could be decreased at deep nanoindentation, i.e., lower density of excess free volume. On the other hand, size effects on plastic deformation have been largely reported in metallic glasses in which plastic flow is facilitated at the nanoscale [8,9,10,11,12], which suggests a better atomic mobility. Qualitatively, the enhanced creep deformation at shallow depth under Berkovich indenter could be explained. Generally, the length scale of the region that suffered yield stress was approximately 3~5 times that of pressed depth under the Berkovich indenter. Once the deformation zone was beyond several hundreds of nanometers, density of shear bands could be stable and size effect would be insignificant on plastic deformation. Therefore, the invariable creep strains at deep locations could be expected under the Berkovich indenter.

Creep strains under spherical indenters were calculated by $0.2\left(\alpha -\alpha_{0}\right)/R$, where *α* and *α*_0_ are the contact radii at the beginning and ending of holding stage, respectively. Figure 5b depicts the correlation between creep strain and holding strain for spherical indenters. Due to the difficulty of precisely computing creep strain at 150, 250 and 500 nm under 0.6 µm spherical indenter, these points were not included. Elastic and plastic holdings were divided for all the spherical indenters, upon the solid and hollow tags. At elastic holdings, creep behaviors clearly exhibited two distinctive features. Creep strains were insignificant (lower than 0.1%) and nearly unchanged with increasing holding strain within the holding range between 0% and 2% (defined as first stage). While for the elastic holding larger than 2% (defined as second stage), creep strain was linearly increased with initial holding strain. Creep strain was rapidly increased from about 0.1% to 0.5%, as the holding strain increased from 2% to 5%. The creep deformation at the elastic region was tightly correlated with initial holding strain and independent of indenter size. Nanoindentation deformation at the first stage could be regarded as purely elastic (less than the 2% elastic limit) from the perspective of nature of metallic glass. Therefore, it is rational that creep flow hardly occurred under elastic holding at room temperature in such a short duration (compared to conventional creep measurement). In Jang et al.’s work, creep deformation was more pronounced in metallic glass pillars with smaller size under elastic holding [17]. It is reasonable that the intrinsic creep resistance was changed with sample size in Jang et al.’s work, due to the fact that free volume content, flaw density and surface damage were all changed with the thinning process by FIB. In present work, however, structural configuration was hardly changed in the elastic region under various spherical indenters. Thus creep strain at elastic holding was tightly tied to the intrinsic creep resistance of as-prepared La-Co-Al film, rather than deformation zone beneath indenter. At the second stage, creep feature can be explained by the deceptively elastic deformation under nanoindentation. Though it was still defined as elastic deformation, the maximum stress has already exceeded yield stress and the irreversible structure agitation, i.e., free volume generation and STZ activation could occur [19]. Both the localized high stress and fertile regions beneath the indenter were beneficial in creep deformation at the second stage. The situations of stress and atomic structure in the two stages were much different, accordingly causing distinctive creep behaviors.

As for plastic holdings, a sudden increase of creep strain can be observed, with the transition of holding strain from elastic to plastic for each spherical indenter. On the plastic holding, creep strain was also increased with holding strain, which is normally expected [33,34]. Creep strains increased from about 0.19% to 0.26% and 0.36% to 0.64% as the holding strains increased from 2.8% to 5.6% and 4.3% to 8.7% for 9.8 µm and 2.95 µm indenters, respectively. While under 0.6 µm indenter, creep strain quickly increased from 0.84% to 1%, as holding strain only increased from 5.4% to 6.7%. Obviously, creep strain was increased slower under larger spherical indenter. The most arresting creep feature at plastic holdings was that creep strain largely relied on indenter size. As an illustration, creep strains were 0.84%, 0.44% and 0.26% at the holding strain ~5.5% under 0.6, 2.95 and 9.8 µm indenters respectively (the corresponding holding depths were 60, 150 and 500 nm respectively). Combined with creep features under Berkovich indenter in Figure 5a, a strong nanoindentation size effect on creep resistance at plastic holding validated that creep deformation was more pronounced in smaller zones suffering the same holding strain. The present creep features at plastic holding effectively enrich Jang et al.’s work about size effect on creep deformation at elastic holding [17].

The present creep feature under nanoindentation was close to conventional creep behavior. Hence it has merits to estimate strain rate sensitivity (SRS), in order to reveal the creep mechanism of metallic glassy film and its correlation with nanoindentation length scale. The value of SRS exponent *m* can be evaluated via:

(4) $$m=\frac{\partial \ln\sigma }{\partial \ln\dot{\varepsilon }}$$

For a standard Berkovich indentation process, the strain rate during the holding stage can be calculated as:

(5) $$\dot{\varepsilon }=\frac{1}{h_{c}}\frac{dh_{c}}{dt}$$

In a spherical-tip indentation process, the strain rate during the holding stage can be calculated as:

(6) $$\dot{\varepsilon }=\frac{1}{\sqrt{A}}\frac{d\sqrt{A}}{dt}$$

where *A* is the contact area, equal to πRh at elastic region and $2\pi Rh_{c}$ at plastic region. The creep flow stress σ can be obtained from the mean pressure $P_{m}$ beneath indenter via Tabor’s mode, $P_{m}$ = 3*σ* [35]. At elastic region under spherical indenters, $P_{m}=\frac{P}{\pi Rh}$. In the plastic region, the mean pressure is also defined as hardness, which is $H=\frac{P}{2\pi Rh_{c}}$ for spherical tip and $H=\frac{P}{Ch_{c}^{2}}$ for a standard Berkovich indenter, where *C* is the tip area coefficient for Berkovich indenter and was rectified upon testing on standard fused silica, equal to 24.3 here. The flow stress and strain rate on holding stage could be estimated from the slope of the fitted linear curve as depicted in Equation (1). The SRS value *m* was determined by linear fitting the log–log correlation between flow stress and strain rate.

**Figure 6:**
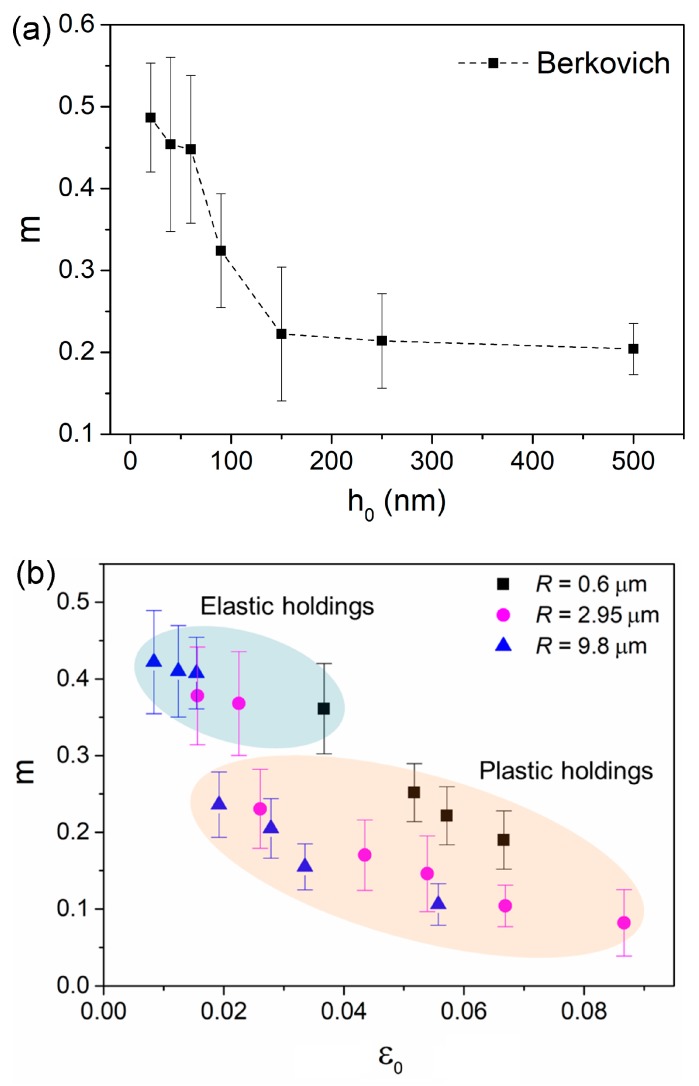
(**a**) The correlation between *m* and holding depth for the Berkovich indenter. (**b**) The correlation between *m* and holding strain for spherical indenters.

Figure 6a shows the estimated *m* and its correlation with holding depth for the Berkovich indenter. SRS was decreased from about 0.5 to 0.22 as holding depth increased from 20 to 150 nm, and then tended to be stable around 0.2. The SRS was apparently higher at the holding depths below 60 nm, which was consistent with Wang et al.’s report of a Zr-based metallic glassy film [22]. Figure 6b shows the correlation between *m* and holding strain for spherical indenters. Approximately, the values of SRS could be divided into two regions relying on deformation manner, i.e., elastic holding and plastic holding, as depicted by different colors. At the “elastic region”, *m* was slightly decreased with holding strain from 0.42 to 0.37. As holding strain increased to the “plastic region”, *m* was dramatically dropped. Further, *m* falls in the range between 0.24 and 0.08, as holding strain increased from about 2% to 8.8%. It should be mentioned that the holding strains of 1.9%, 2.6% and 5.2% for 9.8, 2.95 and 0.6 µm indenters at “plastic region” were actually right below the critical strain at first pop-in event for each indenter. As mentioned earlier, these locations suffered deceptively elastic deformation, in which the stress state was insufficient for shear banding initiation, while enough to arouse plastic unit (free volume and shear transformation zone).

The value of strain rate sensitivity *m* or stress exponent *n* (*n* = 1/*m*) is widely used as an indication of creep mechanism in crystalline alloy or metals. For example, dislocation move is dominating in creep flow as *m* falls in the range between 0.1 and 0.3. In metallic glasses, free volume generation and annihilation, shear transformation zone (STZ) evolution and atomic diffusion (under elastic contact) are thought to be the possible creep mechanisms [23]. While the relationship between *m* and creep mechanism in metallic glass is still inconclusive. For creep deformation under Berkovich indenter, STZ evolution might be the main creep mechanism due to the high holding strain. For the high *m* ~0.45 at 20–60 nm holdings, it could be caused by tip bluntness effect, so that artificial error was inevitable when calculating strain rate and flow stress at such shallow depths. It was unreasonable to regard the great increase of *m* as a transition of creep mechanism. The length scale of a STZ was in the magnitude of 1–2 nm (STZ size was about 1–8 nm^3^) in metallic glasses [36]. The stress state and space beneath the Berkovich indenter could meet the requirement of STZ evolution even at 20 nm on the holding stage (localized shear banding might be suppressed at such shallow depth due to size effect on plastic deformation). SRS of ~0.2 at deep nanoindentation could be deemed as the characteristic value for STZ evolution in creep deformation under Berkovich indentation.

For spherical nanoindentation creep, the distinction of SRS values under “elastic holding” and “plastic holding” could be attributed to the different creep mechanisms. Under purely elastic holdings, STZs were unable to be activated. In this scenario, atomic diffusion between indenter and the contact surface and migration of pre-existed free volume carried creep deformation. While under plastic holding, STZ evolution was dominating in creep flow. The *m* range between 0.24 and 0.08 was much more comparable to that under the Berkovich indenter, indicating the same creep mechanism. On the other side, the phenomenon that *m* decreased with holding strain under plastic holdings could be qualitatively explained upon the enlargement of STZ size at deep nanoindentation [20,27]. Qualitatively, we explained the creep mechanism at elastic plastic holdings in metallic glassy film by SRS, while the true situation of creep flow could be more complicated. In future, atomic force measurement (AFM) on the residual morphology of nanoindentation before and after holding stage could be useful to understand the creep deformation in metallic glass [37].

## 4. Conclusions

In summary, nanoindentation size effect on creep deformation of a La-based metallic glassy film was investigated at room temperature using different indenters. The total creep displacements were nearly linearly increased with holding depth and weakly dependent on indenter type. At a given holding strain ~7.1% under Berkovich indenter, creep resistance was enhanced with increasing holding depth. At the elastic region under spherical indenters, creep deformation was merely dependent on holding strain. At the plastic region, creep deformation was more pronounced at higher holding strains and smaller indenter tips. A strong nanoindentation size effect on creep resistance was validated upon changing holding depth and indenter size. The estimated strain rate sensitivities (SRS) were decreased at first and then tended to be stable with increasing holding depth and holding strain. SRS values detected in purely elastic holdings were around 0.4, which could be correlated with atomic diffusion and free volume migration. And SRS values were approximately between 0.24 and 0.08 for plastic holdings, which indicated that evolution of the shear transformation zone (STZ) was dominating in creep deformation.
